# Perceived barriers and factors influencing uptake of breast cancer screening among women: a population-based cross-sectional study

**DOI:** 10.1038/s41598-024-62218-5

**Published:** 2024-05-29

**Authors:** Angelina A. Joho, Mwajuma B. Mdoe, Theresia J. Masoi, James J. Yahaya

**Affiliations:** 1 Department of Clinical Nursing, School of Nursing and Public Health, University of Dodoma, Dodoma, Tanzania; 2 Department of Pathology, School of Health Sciences, Soroti University, Soroti, Uganda

**Keywords:** Breast cancer screening, Barriers, Uptake, Cancer, Risk factors

## Abstract

Breast cancer (BC) screening plays a major role in the prevention of BC through early detection and timely treatment. This study aims to determine the level of uptake of BC screening and associated factors. A community-based analytical cross-sectional study was conducted in Dodoma City, Tanzania from July to December 2020. The study included women aged 8 years and above without a known history of breast cancer. Multivariable logistic regression was used to determine the socio-demographic factors associated with BC screening. *P* value < 0.05 was considered significant. A total of 354 study participants were included in the present study. The mean age of participants was 31.0 ± 11.8 years. The majority of study participants (67.5%, n = 239) were aware of BC screening. However, only (35.3%, n = 125) reported to have ever practised BC screening. Breast self-examination was the most (16.4%, n = 58) frequently used method for BC screening among study participants. Lack of knowledge of all methods of BC screening was the barrier that was perceived by the vast majority (60.2%, n = 213) of the study participants. Having low family income was the only predictor of failure to practice BC screening. In this study, most of the women were aware of BC, however, few of them had undergone breast cancer (BC) screening at the time of the interview. The study also found that the main barrier to BC screening was the lack of knowledge about BC among the study participants. Immediate measures are necessary to increase women's awareness of BC. Such as community sensitization on the importance of screening, can help improve the uptake of BC screening and the early detection of BC.

## Introduction

Breast cancer (BC) is the leading cause of cancer-related deaths among women globally and its prevalence is on the increase despite stringent measures that are taken in various countries for its mitigation^1–3^. In 2018, it was reported that 15.3% (over 2 million) of all cancer cases were related to BC and also a total of 685,000 cancer-related deaths among women were due to BC^2^. Additionally, the global annual incidence rate for BC has been reported to be 5.8% ^4^. Considering the burden of BC in sub-Saharan Africa, there is underreporting of BC in this region due to a shortage of cancer registries that help in providing cancer-related data. This fact is evidenced by the noticeably disproportionate mortality of BC concerning the low reported burden compared to developed countries^5^. According to 2020 Global Can Data 186,598 BC cases were reported in Africa with 87,787 BC-related deaths^6^.

In Tanzania, BC continuously be a devastating disease and remains the second in terms of prevalence and mortality among women. In 2018, it was estimated that 337,000 cases of BC were newly diagnosed with 1303 BC-related related deaths^7,8^. It is projected that by the year 2040, both the incidence and mortality of BC in Tanzania will escalate by more than 120%^9^. The data provided concerning the prevalence and incidence of BC in Tanzania do not reveal the actual burden of the disease because most of the studies done in Tanzania are clinicopathologic and only a few of the studies address the epidemiological aspects of the disease in the country, this contributes greatly to underreporting of prevalence, incidence rate, mortality rate and many other epidemiological parameters.

BC screening is of paramount importance in providing secondary prevention of diseases. However, in Tanzania, this is still a challenge due to the substantial lack of BC screening. The only available screening centre at the referral and consultant hospitals do not provide wide coverage for BC screening in the country. This contributes to the late detection of BC among patients^10^. The reported level of awareness on various issues related to BC among women in most of the developing countries including Tanzania is disproportionate to the level of uptake of BC screening in most developing countries. This is attributed to several factors including having negative perceptions and barriers which both together limit the accessibility of BC screening services for most women. A few studies done in developing countries have reported significantly low uptake of BC screening, for example studies done in Namibia, Ghana, Kenya and Burkina Faso reported 23.1%^11^, 51.9%^4^, 12%^12^, and 7.5%^11^, respectively.

The uptake of BC screening is influenced by various factors including sociodemographic characteristics, sociocultural factors, health-seeking behaviour, health insurance, knowledge of BC risk factors, and obstetric factors like the number of living children^11,13–15^. Data reporting on BC screening among women in Tanzania is scanty despite the high prevalence, incidence rate and mortality of the disease; this is due to a lack of comprehensive and accessible nationwide BC screening programmes. Therefore, this study aims to assess the prevalence of uptake of BC screening and factors associated as well as barriers **to** BC screening.

## Materials and methods

### Study design and area

This was a cross-sectional analytical community-based study using a quantitative approach. The study was conducted in Dodoma City which is located in the Dodoma region for six months (July-December 2020). According to the 2022 Tanzania Housing and Population Census, the estimated population of the Dodoma region was 3,085,625 of whom 1,512,760 and 1,572,865 were males and females, respectively with a fertility rate of 12.2%^16^, while the number of women with the possibility of giving birth (15–49 years) in the Dodoma region was 5.1%.

### Study population

This study included women from different streets in Dodoma city. The inclusion criteria for the study participants were: all women aged 18 years and above, residents of the selected study site for at least 1 year, without having breast cancer, and agreed voluntarily to participate in the study. All women who were very sick, and mentally ill and those who refused to participate in the study were excluded.

### Sample size and sampling technique

The sample size was estimated using the formula for calculating the prevalence of a single population established by Kish Leslie^17^ as was applied in the previous study considering the following assumptions: Standard normal variables (z score) of 1.96 at 95% confidence interval, proportion (p) of women who had BC screening of 51.9% in Ghana^4^, and 5% level of significance (α = 0.05). The final sample size was adjusted for a non-response rate of 10% and the total sample size estimated was 354. Dodoma city was selected conveniently from seven districts of the Dodoma region. This is because Dodoma is one of the fastest-growing cities in the country, with a high rate of urbanization. This has resulted in a heterogeneous population consisting of people who have migrated from villages to the city. Households were selected using systematic random sampling from which the study participants were obtained. The researchers decided to go on right or left from the starting point. Then the study participants from the households were selected using a simple lottery replacement random sampling method. Pieces of paper were cut and labelled with numbers 1 and 2 and placed in a container which was tightly covered. The container was shaken vigorously to allow the mixing up of the pieces of paper. The container was opened and pieces of paper were poured on the surface to allow the study participants to pick. Those who selected number 2 were recruited in the study.

### Definition of variable

BC screening uptake was considered a categorical variable and responses were dichotomized into “yes” and “no”. All study participants who responded “yes” to the question which was asking “*Have you ever had BC screening”?* to any of the four screening methods (BSE, CBE, Mammography and Ultrasound) were considered to have screened for BC. Additionally, other items were included in measuring BC screening such as “Have you ever heard about BC screening”.

The perceived barrier is the estimation of the level of challenge of social, personal, environmental, and economic obstacles to a specified behavior such as the BC screening procedure.

Factors influencing the uptake of breast cancer screening are attributes that can lead a woman to decide on doing BC screening.

### Data collection and research tool

A structured interviewer-administered questionnaire was used for data collection. The questionnaire was adapted from a previous study^18^ and modified to make it easily understandable including translating it into Swahili local language. A person who was fluent in both English and Swahili was used to translate the questionnaire. To ensure the validity of the questionnaire, we analyzed its internal consistency using Cronbach’s alpha coefficient^19^. We pretested the questionnaire before and after translation among 30 women from a different site. We modified some of the questions that were not clear. We validated the questionnaire based on a Cronbach’s alpha value of ≥ 0.7^19^. In this study, we found Cronbach’s alpha of 0.714 and 0.823 for perceived barrier and practice of breast cancer screening, respectively, which shows that the questionnaire is reliable.

There were three parts in the questionnaire which consisted of sociodemographic characteristics, practices of BC screening, and barriers for BC screening. The face-to-face interviewer method was used to collect the required data. Four research assistants (registered nurses) with experience in data collection were involved in the process of data collection. To avoid participants sharing information, the interview was done at a bit far distance. Approximately, it took 15 to 20 min for one participant.

### Data analysis

Data were analysed using SPSS software version 25.0. Descriptive statistics were used to summarize both categorical and continuous variables in frequencies and percentages and mean ± standard deviation (SD), respectively. Inferential statistics involved the use of binary logistic regression analysis in determining the predictors of BC screening. All variables with *p* ≤ 0.2 in univariate analysis were fitted into multivariable analysis for controlling confounding factors. Odds ratios (ORs) at 95% confidence interval (CI) were determined to assess the predictability of the independent variables. A two-tailed *p* < 0.05 was considered statistically significant.

### Ethical approval

Ethical approval from the Research Ethical Committee of the University of Dodoma was obtained prior to data collection. All the methods were performed in accordance with relevant institutional guidelines and regulations.

### Informed consent

Written informed consent was obtained from all study participants.

## Results

### Sociodemographic characteristics of the study participants

The social demographic characteristics of the study participants are shown in Table 1. A total of 354 study participants’ data were analysed in the present study. The mean age of the study participants was 31.0 ± 11.8 years (range: 18–85 years). The majority 72.3% (n = 256) of study participants were in the age group between 18 and 34 years. Almost half of the study participants 45.5% (n = 161) had primary education. Also, the vast majority 62.7% (n = 222) of the study participants had a family income of less than 1.4 USD (100,000 TZS) per day. Additionally, over half 57.3% (n = 203) of the study participants were married.

**Table 1:** Sociodemographic characteristics of the study participants (N = 354).

Variable	Frequency (n)	Percentage (%)
*Age (years)*		
18–34	256	72.3
35–49	67	18.9
50–85	31	8.8
*MBI (kg/m*^*2*^*)*		
Underweight	5	1.4
Normal	222	62.7
Overweight	97	27.4
Obesied	30	8.5
*Education level*		
Informal	37	10.5
Primary	161	45.5
Secondary	103	29.1
Tertiary	53	15.0
*Economic activities*		
Employed	43	12.1
House wives	138	39.0
Petty traders	173	48.9
*Family income (TZS)*		
< 100,000	222	62.7
100,000 – 500,000	116	32.8
> 500,000	16	4.5
*Marital status*		
Married	203	57.3
Single	103	29.1
Divorced or separated	19	5.4
Cohabiting	14	4.0
Widow	15	4.2
*Religion*		
Christian	220	62.1
Muslim	118	33.3
Traditional	16	4.5

### Awareness and uptake of BC screening among study participants

Concerning awareness of BC screening among participants, the majority 67.5% (n = 239) of the study participants reported being aware of BC screening (mean = 13.2, 95% CI = 12.8–13.7). Almost one-third 35.3% (n = 125) of the study participants reported they had ever screened for BC (mean = 35.3, 95% CI = 30.3–40.3). The most frequently used method for BC screening was breast self-examination, which comprised 16.4% (n = 78), followed by clinical breast examination which accounted for 14.7% (n = 52). Mammography was the least 1.4% (n = 5) used method for BC screening in this study. Of those who reported having had BC screening, most of them 19.5% (n = 69) could have BC screening at any time and only 8.8% (n = 31) of the study participants had BC screening by using breast self-examination method every month for women who aged ≥ 20 years, which is the recommended time interval for BC screening^20^. Furthermore, it was found that those who practised breast self-examination, only (n = 10, 2.8%) were found to perform breast self-examination between 2 and 3 days after menstruation, which is recommended for women who are still getting menstruation and regularly on a monthly bases for menopausal or postmenopausal women^20^ (Table 2).

**Table 2:** Awareness and uptake of BC screening among study participants (N = 354).

Variables	Frequency (n)	Percentage (%)
*Awareness of BC screening*		
Aware	239	67.5
Not aware	115	32.5
*Screening for BC*		
Screened	125	35.3
Never screened	229	64.7
*The method used for BC screening*		
Breast self-examination	58	16.4
Clinical breast examination	52	14.7
Mammography	5	1.4
Ultrasound	10	2.8
None	229	64.7
*Frequency of BC screening*		
After 3 months	10	2.8
After 6 months	31	8.8
After a year	15	4.2
Any time	69	19.5
Never	229	64.7
*The proper time for practising breast self-examination*		
3 to 5 days after menstrual period	10	2.8
3 to 5 days before menstrual period	11	3.1
A few days before the menstrual period	6	1.7
Any time within a month	327	92.4

### Perceived barriers to BC screening among study participants

Table 3 shows the perceived barriers to BC screening. Lack of knowledge of BC was the barrier that was perceived by the vast majority 60.2% (n = 213) of the study participants. Lack of money was the second most 52.5% (n = 186) common perceived barrier by the study participants. Lack of privacy during BC screening was the least 16.4% (n = 58) perceived barrier for BC screening among participants.

**Table 3:** Perceived barriers to BC screening among study participants (N = 354).

Assessed barriers	Yes responses	
	Frequency (n)	Percentage (%)
Pain during screening	118	33.3
Chaos or disgrace during screening	59	16.7
Lack of money	186	52.5
Lack of health insurance	159	44.9
Lack of knowledge of breast cancer	213	60.2
Lack of trust from healthcare workers	86	24.3
Language barrier on breast cancer	104	29.4
Long distance from healthcare facilities	139	39.3
Fear of death after being diagnosed with breast cancer	110	31.1
Not having BC	161	45.5
Lack of privacy during BC screening	58	16.4
Not being comfortable	60	16.9

### I. Multivariable regression analysis for factors associated with BC screening among study participants

After controlling for confounders (age, level of education, marital status, religion, and awareness of breast cancer) in multivariable regression analysis, only family income remained statistically significantly associated with BC screening. Study participants with family income less than 100,000 TZS per month equivalent to less than 1.4 USD per day had a 73% significantly less chance of BC screening (95% CI = 0.08–0.93, *p* = 0.038). Likewise, study participants who had family income between 100,000 and 500,000 TZS per month had 75% significantly less chance of BC screening (95% CI = 0.07–0.88, *p* = 0.031). Religion showed marginal statistical significance with BC screening in which study participants who were either Christians 3.97 (95% CI = 0.86–18.38, *p* = 0.078) or Muslims 4.44 (95% CI = 0.94–20.89, *p* = 0.060) had increased odds of BC screening compared with study participants that had traditional beliefs even though the difference did not reach statistical significance.

Another variable that showed marginal statistical significance with BC screening was level of education, whereby study participants with primary education had 53% less chance of BC screening (95% CI = 0.21–1.05, *p* = 0.064). Other variables including age, marital status, and awareness of BC screening although did not show a statistical association with BC screening, still they showed a trend of increased odds towards BC screening (Table 4).

**Table 4:** Multivariable regression analysis for sociodemographic factors associated with BC screening among study participants.

Variable	*P*	COR (95% CI)	*P*	AOR (95% CI)
*Age (years)*				
18–34	0.611	1.24 (0.55–2.80)	0.973	0.98 (0.37–2.60)
35–49	0.142	1.98 (0.80–4.94)	0.491	1.43 (0.52–3.94)
≥ 50		Ref.		Ref.
*BMI (kg/m*^*2*^*)*				
Underweight	0.405	0.38 (0.04–3.78)		
Normal	0.382	0.71 (0.32–1.54)		
Overweight	0.826	1.10 (0.48–2.6)		
Obesity		Ref.		
*Education level*				
Informal	0.084	0.46 (0.19–1.11)	0.293	0.57 (0.19–1.64)
Primary	0.01	0.43 (0.23–0.82)	0.064	0.47 (0.21–1.05)
Secondary	0.055	0.52 (0.26–1.02)	0.203	0.60 (0.27–1.32)
Tertiary		Ref.		Ref.
*Economic activities*				
Employed	0.427	1.32 (0.67–2.59)	0.72	0.86 (0.38–1.95)
Housewives	0.147	0.70 (0.44–1.30)	0.32	0.77 (0.47–1.28)
Petty traders		Ref.		Ref.
*Family income (TZS)*				
< 100,000	0.003	0.17 (0.05–0.55)	0.038	0.27 (0.08–0.93)
100,000 – 500,000	0.003	0.16 (0.05–0.54)	0.031	0.25 (0.07–0.88)
> 500,000		Ref.		Ref.
*Marital status*				
Married	0.131	2.71 (0.74–9.91)	0.221	2.41 (0.59–9.89)
Single	0.51	1.57 (0.41–5.96)	0.648	1.42 (0.32–6.38)
Divorced or separated	0.451	1.85 (0.38–9.08)	0.782	1.27 (0.23–7.00)
Cohabiting	0.349	2.22 (0.42–11.83)	0.410	2.14 (0.35–13.01)
Widow		Ref.		Ref.
*Religion*				
Christian	0.084	3.77 (0.84–17.02)	0.078	3.97 (0.86–18.38)
Muslim	0.055	4.47 (0.97–20.59)	0.060	4.44 (0.94–20.89)
Traditional		Ref.		Ref.
*Awareness*				
Aware	0.081	1.64 (0.94–2.86)	0.326	1.36 (0.74–2.48)
Not aware		Ref.		Ref.

## Discussion

Breast cancer screening helps early detection, particularly in settings where treatment modalities for breast cancer are still an enigma. This greatly contributes to the red flag of poor clinical outcomes of the patients with BC as a result of late detection of the disease. We aimed to determine the level of uptake of BC screening and factors associated as well as barriers to BC screening in Dodoma city which is a rapidly growing city in Tanzania. The main findings in this study include a low level of uptake of BC screening and the association of low family income with low uptake of BC screening. Additionally, lack of knowledge of BC among study participants was reported to be the major barrier to BC screening.

Although BC screening has been found to significantly reduce the burden of BC and also improve clinical outcomes of patients through early detection, the uptake of BC screening in developing countries is still significantly low. This contributes to late detection and poor outcomes for patients with BC in most developing countries. The uptake of BC screening of 35.3% obtained in this study was similar to the 33.8% uptake of BC screening which was reported in a study done by Emma et al. in Tanzania^18^ but lower than 51.9% of uptake of BC screening reported in Ghana ^4^. However, other studies done in Indonesia, Nigeria, and Saudi Arabia reported significantly low levels of uptake of BC screening 18.7%^15^, 9.9%^21^, 16.2%^22^, respectively. Despite Ghana and Tanzania being low-middle income countries, the prevalence of modern FP use in Ghana was associated with the difference in the age of study participants, the Ghanaian study included the population of young reproductive age, such women are more likely to have more knowledge.

One of the reasons for the discrepancy in the uptake rates for BC screening observed in various studies is due to the difference in methods of BC screening used, for example, studies which used a single method of BC screening, particularly mammography have reported significantly low rate of uptake of BC screening^21^, this may be explained by the fact that the coverage of the availability of mammography in most of developing countries is still a challenge which prompts utilization of other methods of BC screening. The disproportion of awareness of BC and uptake of BC screening observed in different studies helps to explain the existing discrepancy in the uptake rates of BC screening in various studies. There is a larger number of studies which have reported a high level of awareness of BC screening among women in both developed and developing countries which is not reflected by the level of uptake of BC screening. This is evidenced by high level of awareness of BC screening in this study, however, the uptake rate of BC screening was significantly low similar to the findings in the studies done by Emma et al. and Alshahrani et al.^18,23^. Socio-cultural factors and beliefs could also influence the uptake of BC screening among participants in a given study. In countries that exercise strict social and cultural norms for example interaction of women with men has been found to have a low uptake rate of BC screening despite being developed^22,24^.

Various sociodemographic factors have been found to influence the uptake of BC screening among women. In this study, it was found that women who had low family income had reduced odds for BC screening. This indicates that women in families with high family income have high chance of screening for BC. Other studies have also reported a positive association between low family income and reduced uptake of BC screening^15,22,25–27^. Good family income plays a major role in facilitating BC screening among women through various ways including transport, access to media such as new paper, internet and television, and attending seminars among many others^28,29^. Additionally, it has been found that good income is linked with good socioeconomic status such high level of education, health insurance, and marital status which all together influence positively BC screening^28,30^.

The association of formal education with BC screening among women has been reported by many studies albeit few studies which have reported a converse relationship between the two variables. In this study women with education level ranging from primary to tertiary had increased odds of BC screening compared to women with informal education. This was also observed in the studies of Ayanore et al.,^29^, Opoke et al.^31^, Mousavi et al.^32^, and Islam et al.^33^. High level of formal education which is one of the indicators of high socioeconomic status has a positive influence on health-seeking behaviour. Women who acquire formal education in their lifetime tend to increase the possibility of engaging themselves in various health-seeking behaviours including BC screening, unlike women who are not privileged to obtain formal education in their life. This is reflected by low rate of uptake BC screening among women residing in rural areas where there is a challenge of getting formal education^29,34,35^.

Awareness is a standalone factor in prevention of diseases including BC through screening. Increased awareness of BC significantly improves early detection of BC which in turn helps to improve the clinical outcome of patients with BC. In this study, it was observed that women who were aware of BC were 3 times more likely to embark on BC screening compared with women who were not aware of BC. This was also found in the studies of Ngani et al.^27^, Solika et al.^15^, and Abiyu et al.^36^. In any community globally, it is highly stipulated that raising awareness among people in a given community is mandatory as a primary prevention approach of diseases that is why the World Health Organization (WHO) as well as Centre for Disease Control (CDC) and many other global health organizations emphasis on the importance of these disease prevention approach^37,38^.

Barriers to BC screening contribute largely to inadequate screening practices among women especially in developing countries. This leads to delay early detection of BC and contribute to increased morbidity and poor prognosis. Early detection of BC aids in timely presentation of symptomatic BC for treatment and down-staging^39^. For both qualitative and quantitative studies done previously, it has been clearly shown that lack of awareness and knowledge of BC among women is associated significantly with failure of most women to screen for BC globally^11,22,28,40^ which is similar to the finding in this study. This experience is even more common in developing countries such as Tanzania where provision of public health is still a challenge. The observed high percentage of women that lack awareness and knowledge of BC in developing countries is attributed to lack of formal education among women.

Challenges with family income and not having BC were other factors that were perceived as barriers to BC screening among study participants in the present study. Similarly, other two previous studies^29,41^ reported that low family income hinders women from accessing health facilities for BC screening, having ability to own sources of information such as television^22^. Studies have shown that, women who perceive that have no BC do not see the importance of going or practising BC screening^42^. Others fear of BC screening because they are afraid of receiving a positive diagnosis^43^. It is imperative to have a holistic approach in improving means of raising awareness and knowledge of BC among women so as to change their minds towards BC screening.

Our study had some limitations. One of the limitations was that in this study it was not possible to analyze the uptake of BC screening for each screening method because some of the methods for example, mammograph and ultrasound were used by very few study participants. This limited us to perform regression analysis and for that reason we could not determine the factors associated with uptake with BC as per specific screening methods. Another limitation was that the uptake of BC screening was self-reported which is usually associated with information bias. Selection of the study subjects was by using convenience sampling method, this might have contributed to selection bias. Also, our findings lack generalizability due to the reason that, participants were taken from one area making the sample not representative of the whole country.

## Conclusion

This study revealed that the majority of women were aware of BC. However, few of them reported to practise BC screening by the time when they were interviewed. Additionally, it was observed that lack of knowledge of BC among study subjects was the main barrier towards BC screening. From these findings, it can be learned that the rate of uptake of BC screening is still quite low and therefore, immediate measures including increasing the level of women’s awareness of BC to improve the uptake of BC screening and increase early detection of BC as well. We also recommend further study that will be more objective with a valid and sensitive instrument to enable more analytical process with a larger population of respondents.

## Data Availability

The dataset used for this study can only be accessed upon reasonable request from the corresponding author.
